# Antioxidant and Antibacterial Activities of the Leaf and Stem Extracts of *Combretum molle* (R. Br. ex G. Don.) Engl. & Diels

**DOI:** 10.3390/plants12091757

**Published:** 2023-04-25

**Authors:** Myuri Parusnath, Yougasphree Naidoo, Moganavelli Singh, Farzad Kianersi, Yaser Hassan Dewir

**Affiliations:** 1 School of Life Sciences, University of KwaZulu-Natal, Westville Campus, Private Bag X54001, Durban 4000, South Africa; 2 School of Environmental Sciences, University of Guelph, 50 Stone Road East, Guelph, ON N1G 2W1, Canada; 3 Plant Production Department, College of Food and Agriculture Sciences, King Saud University, Riyadh 11451, Saudi Arabia

**Keywords:** medicinal plants, radical scavenging activity, ferric-reducing antioxidant power, agar well diffusion

## Abstract

Medicinal plants offer reasonable and accessible alternatives to synthetic drugs and are often devoid of the adverse side effects, toxicity, and pathogenic resistance associated with synthetic medicine. *Combretum molle* has been utilized in African traditional medicinal practices and purportedly contains bioactive compounds with medicinally beneficial effects. This study investigated the hexane, chloroform, and methanol leaf and stem extracts for their antioxidant properties using the 2,2′-diphenyl-1-picrylhydrazyl radical scavenging and ferric-reducing antioxidant power assays. The study additionally analyzed the methanol extracts for their antibacterial activity against Gram-negative *Escherichia coli* (ATCC 25922) and Gram-positive *Staphylococcus aureus* (ATCC 25923) bacteria using agar well diffusion. Relative to the scavenging activity of the ascorbic acid control (79.15 ± 0.63% at 15 µg/mL to 94.61 ± 0.12% at 240 µg/mL), the plant’s radical scavenging activities were exceptionally high in the methanolic leaf and stem extracts (*p* < 0.05), ranging from 94.58 ± 1.10% at 15 µg/mL to 99.22 ± 0.30% at 240 µg/mL and 91.57 ± 1.71% at 15 µg/mL to 99.60 ± 0.20% at 240 µg/mL, respectively, suggesting a strong capacity to donate hydrogen ions. High scavenging activities were additionally observed in the chloroform stem (78.68 ± 1.18% at 15 µg/mL to 98.14 ± 1.22% at 240 µg/mL) and hexane leaf (72.12 ± 4.38% at 15 µg/mL to 89.87 ± 1.50% at 240 µg/mL) extracts (*p* < 0.05). All extracts exhibited poor ferric-reducing abilities in relation to the gallic acid control (100 ± 0.00%) at all concentrations (*p* < 0.05). The leaf and stem extracts exhibited broad-spectrum antibiotic capabilities against both tested strains, with significant activity at higher concentrations (*p* < 0.05). Overall, both the leaf and stem extracts of *C. molle* exhibited similar antioxidant and antibacterial activities. These findings warrant further pharmacological research on *C. molle* for potential drug development.

## 1. Introduction

Early civilizations relied on herbal and traditional medicinal practices to treat their medical conditions [1]. Medicinal plant consumption is currently increasing in both traditional and modern practices [2]. In developing African countries, people from disadvantaged and resource-poor communities benefit from the use of medicinal plants, which are relatively cheaper and more accessible than modern medicines [3,4]. Importantly, some synthetic, modern medicines have been noted to cause adverse side effects [5,6], while others have low potencies against certain pathogens [7,8]. In addition, the indiscriminate use of antibiotics has led to numerous bacterial strains developing resistance to synthetic antimicrobial agents [7,8,9]. Therefore, alternate sources of pharmacologically active compounds are required to effectively treat diseases, resulting in research intensification on medicinal plant species [10]. Plants contain a large array of bioactive phytocompounds [11] that have been used in medical treatment since historical times [12,13]. Several plant species within the pantropical, medicinal family, Combretaceae R. Br., particularly the genus *Combretum* Loefl., have been purportedly used in African traditional medicinal practices to alleviate symptoms and treat disease [14,15,16]. The extracts of one such species, *Combretum molle* (R. Br. ex G. Don; Engl. and Diels [velvet bushwillow]), has been reported to treat illnesses associated with oxidation and bacterial infection [14,17,18]. This species’ medicinal properties are conceivably attributed to several bioactive phytocompounds that interact synergistically to produce these medicinal effects [19]. Previously isolated bioactive compounds that provide *C*. *molle* with its antioxidant activities include gallic acid [15] and punicalagin [20], whereas compounds such as Combretenes A and B [21], mollic acid glucoside [22], combregenin, arjungenin, and combreglucoside [20,23] are known to exert antibacterial effects.

Antioxidants are produced endogenously by mammalian bodies to mitigate free-radical damage and prevent oxidative stress caused by increased reactive oxygen species (ROS) [24,25]. Damage by ROS leads to a loss of cell structure and function, inherently causing disease [10,26]. When ROS production exceeds the body’s natural antioxidant capacity, exogenous sources are needed to combat oxidative damage [27]. A number of synthetic antioxidants have been shown to produce toxic and mutagenic effects, leading to plant-based compounds being investigated for their abilities to scavenge free radicals and reduce their damage [10]. Several species of the genus *Combretum* have been reported to possess antioxidant activities [28,29,30,31]. Koevi et al. [28] showed that ethanolic leaf extracts of *C*. *molle* presented antiradical activity when tested using the 2,2′-diphenyl-1-picrylhydrazyl and nitric oxide radical scavenging assays. Methanol and acetone extracts of the leaves of *Combretum indicum* and *Combretum racemosum*, respectively, were also reported to produce radical scavenging activities [29,30]. Furthermore, a study on the ethanol extracts of *Combretum albidum* revealed that the plant’s ability to reduce oxidative damage was due to elevated superoxide dismutase activity [31].

During bacterial infection, increased ROS production is beneficial in the prevention of the microbial colonization in tissues [8]. However, this ultimately results in an aggravated condition for an infected patient [8]. The adverse side effects of synthetic antimicrobial agents and the increasing resistance of pathogens to current antibiotics is a global health challenge [8,32]. There have been a number of investigations on the antibacterial efficacies of *Combretum* species. Chukwujekwu and Van Staden [33] reported that dichloromethane leaf fractions of *Combretum edwardsii* were highly and moderately effective against *Staphylococcus aureus* (ATCC 11632) and *Escherichia coli* (ATCC 25218), respectively, while ethyl acetate leaf fractions of *Combretum krausii* were more effective against *S*. *aureus* than *E*. *coli*. Burman et al. [7] found that aqueous and ethanolic leaf extracts of *Combretum album* showed significant activity against several bacterial strains, including *Pseudomonas aeruginosa* (MTCC 2453), *Bacillus subtilis* (MTCC 441), and *E*. *coli* (MTCC 739). Acetone, ethyl acetate, and dichloromethane extracts of *C*. *molle* leaves were found to inhibit the growth of *Enterococcus faecalis* (ATCC 29212), *S*. *aureus* (ATCC 29213), *E*. *coli* (ATCC 25922), and *P. aeruginosa* (ATCC 27853) [34].

Despite there being several studies on the bioactivities of the plant’s leaf extracts, there is a paucity of studies conducted on the stem extracts of *C*. *molle*. In order to advance the traditional use of plant extracts into modern drug development, there is a need to critically investigate the biological activities of medicinal plant species. Since various species of *Combretum* have been reported to exhibit antioxidant and antibacterial activities, and due to the plethora of bioactive compounds present within *C*. *molle*, it is imperative to further research and compare this plant’s leaf and stem bioactivities when extracted with various solvents. This study aimed to determine the antioxidant potential of hexane, chloroform, and methanol leaf and stem extracts of *C*. *molle* using the 2,2′-diphenyl-1-picrylhydrazyl (DPPH) radical scavenging and the ferric (Fe^3+^)-reducing antioxidant power (FRAP) assays. Additionally, this study aimed to evaluate the antibacterial efficacy of the methanol leaf and stem extracts against clinical strains of *E*. *coli* (ATCC 25922) and *S*. *aureus* (ATCC 25923) bacteria using the agar well-diffusion technique. To the best of our knowledge, this study is the first report that compares the plant’s DPPH scavenging activity with its ferric-reducing abilities.

## 2. Results

### 2.1. DPPH Radical Scavenging Activity

**Figure 1:**
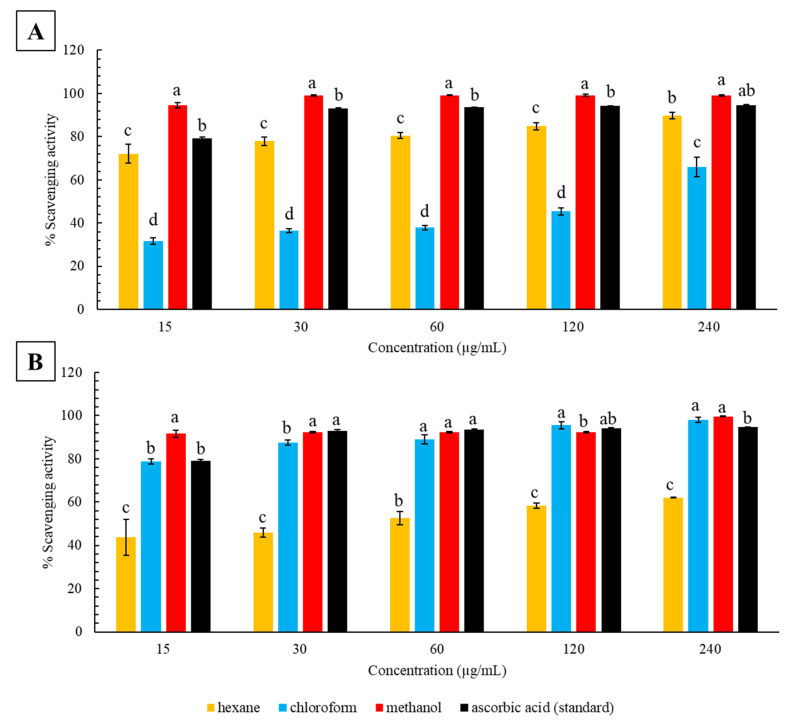
DPPH radical scavenging activity (%) of increasing concentrations (15, 30, 60, 120, and 240 µg/mL) of hexane, chloroform, and methanol extracts of *C*. *molle* (**A**) leaves and (**B**) stems. Values represent the mean ± standard deviation of three replicates. Different letters (a–d) indicate significant differences in scavenging activity between different solvents within each concentration (*p* < 0.05).

The DPPH radical scavenging activity of all extracts (leaves and stems extracted in hexane, chloroform and methanol) of *C*. *molle* were dose-dependent and increased with increasing concentrations (15 to 240 µg/mL) (Figure 1). At each concentration, the scavenging activity of the ascorbic acid standards increased from 79.15 ± 0.63% at 15 µg/mL to 93.00 ± 0.41, 93.62 ± 0.07, 94.18 ± 0.11, and 94.61 ± 0.12% from 30 to 240 µg/mL, respectively. Leaves extracted in chloroform produced the lowest radical scavenging activity at all concentrations (31.69 ± 1.39, 36.51 ± 0.94, 37.88 ± 1.06, 45.39 ± 1.67 and 65.86 ± 4.51%), followed by higher activities in the hexane (72.12 ± 4.38, 77.86 ± 2.01, 80.51 ± 1.42, 84.81 ± 1.75, and 89.87 ± 1.50%) and methanol (94.58 ± 1.10, 98.96 ± 0.28, 99.18 ± 0.24, 99.20 ± 0.57, and 99.22 ± 0.30%) extracts, respectively (Figure 1A). Relative to the standard, the hexane leaf extracts from 15 to 120 µg/mL were significantly lower (*p* < 0.05), indicating moderate antioxidant activity, whereas stronger activity was observed at 240 µg/mL where the extract was comparable to the standard (*p* > 0.05). At all concentrations, the chloroform leaf extracts were significantly lower than the ascorbic acid standards and demonstrated weak-to-moderate scavenging activity with increasing concentration (*p* < 0.05). The methanol leaf extracts were significantly greater than the standard from 15 to 120 µg/mL (*p* < 0.05) and exhibited similarly elevated scavenging activity at 240 µg/mL (*p* > 0.05). Within each concentration, each leaf extract produced significantly different radical scavenging activities based on extraction solvent (*p* < 0.05). Stems extracted in hexane produced the lowest activities (43.73 ± 8.22, 45.96 ± 2.05, 52.53 ± 2.96, 58.19 ± 1.26, and 62.03 ± 0.06%), followed by those extracted in chloroform (78.68 ± 1.18, 87.54 ± 1.28, 89.03 ± 2.06, 95.50 ± 1.66, and 98.14 ± 1.22%) and methanol (91.57 ± 1.71, 92.34 ± 0.39, 92.34 ± 0.32, 92.39 ± 0.26, and 99.60 ± 0.20%), respectively (Figure 1B). However, the stems extracted in chloroform at 120 µg/mL produced significantly greater radical scavenging activity than the methanol extract (*p* < 0.05). When compared to the activity of the control, stems extracted in hexane were significantly lower, suggesting weak scavenging activity (*p* < 0.05). The chloroform extracts were significantly lower than the ascorbic acid control at 30 µg/mL (*p* < 0.05) and comparable at all other concentrations (*p* > 0.05), representing moderate-to-strong activity with increasing concentrations. Scavenging activity of the stems extracted in methanol was significantly greater than the controls at 15 and 240 µg/mL (*p <* 0.05) and comparable at all other concentrations (*p >* 0.05), suggesting strong antioxidant activity at all concentrations. Within each concentration, each solvent produced significant differences in radical scavenging activity (*p* < 0.05), except for the chloroform and methanol stem extracts at 60 and 240 µg/mL (*p* > 0.05). Overall, the leaves extracted in hexane produced significantly higher radical scavenging activities than the stems (*p* < 0.05). In contrast, stems extracted in chloroform demonstrated significantly higher activities (*p* < 0.05). In the methanol extracts, leaves were significantly higher from 30 to 120 µg/mL (*p* < 0.05), whereas at 15 and 240 µg/mL, stem extracts presented significantly higher radical scavenging activities (*p* < 0.05).

For each extract, the concentration of antioxidants needed to scavenge the initial DPPH radicals by 50% are reported in Table 1. Methanol leaf and stem extracts had exceptionally low IC_50_ values of 1.52 × 10^−7^ ± 2.93 × 10^−8^ and 2.46 × 10^−14^ ± 8.53 × 10^−15^ µg/mL, respectively (Table 1). Low IC_50_ values were additionally observed in the ascorbic acid standard, followed by the chloroform stem extract, which additionally presented high scavenging activity, and the hexane leaf extract, which exhibited moderate activity. The chloroform leaf and hexane stem extracts produced elevated IC_50_ values, suggesting poor scavenging capacities. Differences among all extracts were non-significant except for the hexane stem, which displayed significantly higher IC_50_ values (*p* < 0.05).

### 2.2. Ferric-Reducing Antioxidant Power

**Figure 2:**
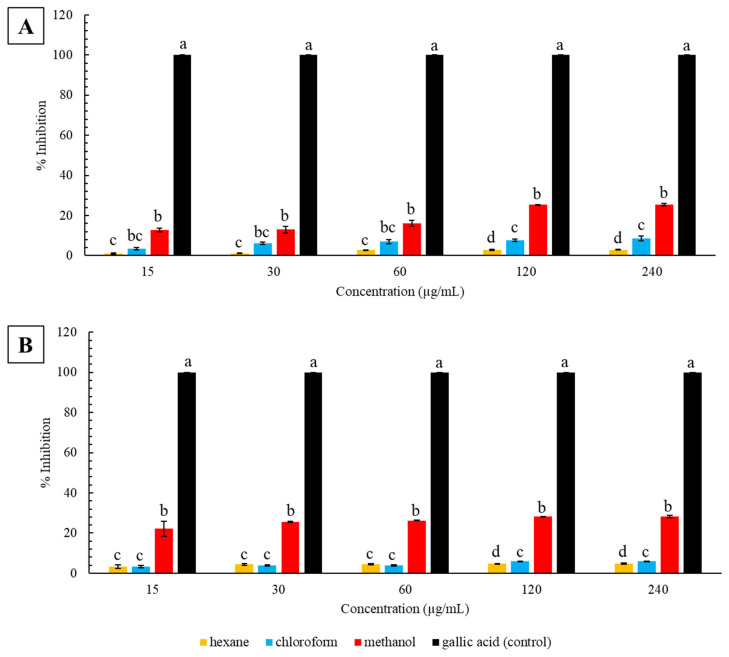
Ferric-reducing antioxidant power (% inhibition) of increasing concentrations (15, 30, 60, 120, and 240 µg/mL) of hexane, chloroform, and methanol extracts of *C*. *molle* (**A**) leaves and (**B**) stems. Values represent the mean ± standard deviation of three replicates. Different letters (a–d) indicate significant differences in inhibition between different solvents within each concentration (*p* < 0.05).

The ferric-reducing abilities of all *C*. *molle* extracts (leaves and stems extracted in hexane, chloroform, and methanol) were dose-dependent and increased with increasing concentrations from 15 to 240 µg/mL (Figure 2). The gallic acid controls had 100 ± 0.00% ferric-reducing abilities at all tested concentrations. The reducing abilities (percent inhibition of oxidation) of the leaf extracts are illustrated in Figure 2A. A low ferric-reducing ability was observed in leaves extracted in hexane (1.03 ± 0.19, 1.11 ± 0.18, 2.70 ± 0.11, 2.74 ± 0.30, and 2.91 ± 0.16%), followed by minor increases in the chloroform (3.51 ± 0.58, 6.13 ± 0.63, 6.99 ± 0.99, 7.65 ± 0.63, and 8.54 ± 1.28%) and methanol (12.77 ± 0.97, 13.01 ± 1.69,16.07 ± 1.59, 25.33 ± 0.13 and 25.37 ± 0.66%) extracts, respectively. Relative to the gallic acid controls, all leaf extracts were significantly lower, indicating poor reducing abilities (*p* < 0.05). Within each concentration, oxidation inhibition for the leaf extracts was significantly different (*p* < 0.05). However, from 15 to 60 µg/mL, inhibition by the chloroform extracts were comparable to the hexane and methanol extracts (*p* > 0.05). The reducing power of the stem extracts are presented in Figure 2B. The stems extracted in hexane had the lowest oxidation inhibition (3.27 ± 0.98, 4.46 ± 0.47, 4.49 ± 0.24, 4.75 ± 0.14, and 4.86 ± 0.32%), followed by the chloroform (3.39 ± 0.62, 3.88 ± 0.38, 3.93 ± 0.21, 5.87 ± 0.28, and 5.98 ± 0.06%) and methanol (22.15 ± 3.73, 25.57 ± 0.37, 26.31 ± 0.15, 28.17 ± 0.16, and 28.22 ± 0.55%) extracts, respectively. However, at 30 and 60 µg/mL, hexane extracts had a higher inhibition than the chloroform extracts. In relation to the inhibition of the gallic acid controls, all extracts were significantly lower (*p* < 0.05), indicative of poor reducing abilities. Within each concentration, inhibition within the stem extracts was significantly different (*p* < 0.05). However, hexane and chloroform extracts were not significantly different from one another from concentrations of 15 to 60 µg/mL (*p* > 0.05). Overall, hexane and methanol stem extracts exhibited significantly higher inhibition compared to leaf extracts (*p* < 0.05). In contrast, the chloroform leaf extracts exhibited significantly higher inhibition from 30 to 240 µg/mL (*p* < 0.05), and similarly higher inhibition at 15 µg/mL (*p* > 0.05).

The concentration of antioxidants in the extracts required to inhibit oxidation by 50% (IC_50_) are presented in Table 2. All extracts produced elevated IC_50_ values ranging from 663.77 ± 74.71 µg/mL in the methanol leaf extract to 8358.53 ± 5514.41 µg/mL in the hexane stem extract. The hexane stem extract was significantly higher than both the methanol leaf extract and the gallic acid control (*p* < 0.05), while the values for all other extracts were similar (*p* > 0.05). The high IC_50_ values indicated low reducing abilities relative to the gallic acid control, which produced a low IC_50_ of 0.53 ± 0.16 µg/mL, indicating high inhibitory activity.

### 2.3. Antibacterial Activity

Based on the antioxidant activities, stems and leaves of *C*. *molle* extracted in methanol displayed higher scavenging activity (*p* < 0.05) than the other extracts and were, hence, chosen for the antibacterial assay. The results of the antibacterial assay revealed that the inhibition of both *E*. *coli* and *S*. *aureus* growth notably increased as the leaf and stem methanol extract concentrations increased from 0.625 to 10 mg/mL (Table 3). No inhibition was observed for the negative controls. Relative to the antibiotic positive controls (10 µg/mL of gentamicin and streptomycin), the inhibition of bacterial growth by the leaf extracts was significantly lower against *E*. *coli* and *S*. *aureus* from 0.625 to 2.5 mg/mL (*p* < 0.05). However, at 5 and 10 mg/mL, the inhibition was similar to the controls, suggesting an effective inhibitory activity against both strains at these concentrations (*p* > 0.05). At 10 mg/mL, *S*. *aureus* exhibited higher susceptibility to the leaf extract than to streptomycin, producing a greater inhibition zone (14.50 ± 1.08 mm) than the antibiotic (12.33 ± 1.25 mm). When compared to the antibiotics, the antibacterial activity of the stem extracts was significantly lower from 0.625 to 5 mg/mL (*p* < 0.05). However, at 10 mg/mL, bacterial inhibition was comparable to the controls, indicating effective inhibitory activity against both *E*. *coli* and *S*. *aureus* (*p* > 0.05). For both strains, the leaf and stem extracts produced similar inhibition zones at all concentrations (*p* > 0.05). However, at 2.5 mg/mL, the leaf extract against *E*. *coli* produced significantly higher antibacterial activity than the stem extract (*p* < 0.05). Furthermore, the leaf and stem extracts exhibited similar activity against both strains (*p* > 0.05), although *S*. *aureus* was significantly more susceptible to the leaf extract at 10 mg/mL than *E*. *coli* (*p* < 0.05).

## 3. Discussion

### 3.1. Antioxidant Activity

The antioxidant abilities of the leaf and stem extracts of *C*. *molle* were assessed using the spectrophotometric DPPH radical scavenging and FRAP inhibition assays. These assays provided insight on the extracts’ abilities to scavenge ROS, reduce oxidative substances, and, thus, inhibit oxidation [35,36]. In this study, the DPPH assay was based on the ability of the *C*. *molle* leaf and stem extracts to donate a hydrogen ion to the DPPH radical, essentially reducing it from 2,2′-diphenyl-1-picrylhydrazyl to 2,2′-diphenyl-1-picrylhydrazine (DPPH-H) [37,38,39]. The data from the assay revealed that a number of the analyzed extracts of *C*. *molle* had significant antioxidant activities, and that higher extract concentrations produced higher activities (Figure 1). The dose-dependent nature of the plant’s antioxidant activity is possibly due to higher amounts of phytochemicals with radical scavenging abilities occurring at higher extract concentrations [40]. At the tested concentrations of 15 to 240 µg/mL, scavenging activity of the methanol leaf and stem extracts were significantly higher or comparable to the ascorbic acid standards, representing strong antioxidant abilities. Similar scavenging activity within the leaves and stems suggests that similar phytochemical constituents responsible for the antioxidant effects were extracted from these organs. Low IC_50_ values are indicative of high antioxidant activity. The IC_50_ values of the methanol extracts supplemented the radical scavenging data with remarkably low values (Table 1). The *C*. *molle* stem extracted in chloroform was additionally found to be effective in scavenging the DPPH free radicals, with activity ranging from moderate to strong as the concentration increased. At higher concentrations (120 and 240 µg/mL), these extracts were more effective than the ascorbic acid controls. Conversely, leaves extracted in chloroform produced low-to-moderate activities, as evident from their correspondingly high IC_50_ values. In addition, the hexane leaf extracts exhibited moderate-to-high scavenging activities, with similar activity to the ascorbic acid standard at 240 µg/mL, while stems extracted in hexane produced low activities. Notably, extracts of differing concentrations produced similar activities. This may be due to the extracts containing a favorable number of phytochemicals responsible for the plant’s antioxidant properties even at lower concentrations and increasing only slightly with increasing concentrations.

The findings from this study are congruent with other analyses on the scavenging ability of *C*. *molle* extracts. Rademan et al. [41] found high DPPH radical scavenging activities in *C*. *molle* leaves and fruit extracted in ethanol, with correspondingly low IC_50_ values of 1.9 ± 0.01 and 5.1 ± 0.05 µg/mL, respectively. Furthermore, Koevi et al. [28] investigated the radical scavenging activity of the plant’s ethanol leaf extracts but obtained a moderate IC_50_ value of 42 ± 0.07 µg/mL. The IC_50_ value of the leaves in the present study was comparatively low, indicating that methanol extracts may be more effective in scavenging free radicals. The high scavenging abilities present in the methanol extracts may be attributed to the solvent’s ability to extract phytochemical constituents such as phenolic compounds and flavonoids, which are associated with producing antioxidant effects [26,40]. Structurally, these compounds comprise an aromatic ring, which enables free radical scavenging by the donation of hydrogen atoms [40,42]. There are various reports of extracts of *C*. *molle* containing phenolic compounds and flavonoids [15,19,28,43], corroborating these findings. In contrast, Ntshanka et al. [19] found that when compared with the acetone, chloroform, and ethanol fractions of the plant’s leaf, methanol fractions presented lower scavenging activities with elevated IC_50_ values. These deviations may be due to variations in location, climate, harvesting season, and extraction methods [44,45]. Nevertheless, methanol extracts of other species in the genus have displayed high radical scavenging activities. *Combretum apiculatum* subsp. *apiculatum* leaves extracted in methanol were reported to produce significant activities, with a half maximal effective concentration of 14.5 ± 0.12 μg/mL [46]. Manga [47] revealed that methanol leaf and root bark extracts of *C*. *racemosum* additionally produced considerably high activities with IC_50_ values of 3.00 ± 0.30 and 2.90 ± 0.40 µg/mL, respectively. The investigation further analyzed the scavenging activities of the methanolic leaf extracts of *Combretum celastroides* subsp. *Laxiflorum,* which demonstrated high activities with an IC_50_ value of 5.00 ± 0.10 µg/mL. Methanol extracts of the leaves of *C*. *indicum* produced an IC_50_ value of 48.87 µg/mL, indicative of moderate scavenging activity [30]. Moreover, *Combretum micranthum* was analyzed for its DPPH scavenging activity. In addition, a hydroalcoholic leaf extract comprising ethanol and water produced an IC_50_ of 2.49 ± 0.53 µg/mL [48]. Effective antioxidant activities from both methanol and ethanol extracts may be attributed to the high polarity of these alcoholic solvents, resulting in the extraction of similar phytochemicals [49,50].

The FRAP assay was used to determine the antioxidant capabilities of the *C*. *molle* leaf and stem extracts by its ability to donate an electron, causing the reduction of potassium ferricyanide (Fe^3+^) to potassium ferrocyanide (Fe^2+^) [51,52]. The results showed that all extracts had low reducing powers, indicative of a low inhibition of oxidation (Figure 2). The extracts were highly ineffective in comparison to the gallic acid control, as substantiated by the elevated IC_50_ values (Table 2). Regardless of the poor inhibition, *C*. *molle* leaves and stems extracted in methanol had a significantly higher reducing power than the chloroform and hexane extracts, corresponding with the findings from the DPPH assay. A recent analysis on ethyl acetate and aqueous fractions of methanol extracts of *C*. *micranthum* leaves revealed high antioxidant capabilities when analyzed using both DPPH and FRAP assays [53]. These findings are in contrast to the data of the present study, which comprises a large variation in antioxidant activities, involving high scavenging activity but low ferric-reducing abilities. This implies that the *C*. *molle* leaf and stem extracts were effective in donating hydrogen ions as illustrated by the reduction of DPPH but may not be effective in donating electrons as shown by the poor ferric-reducing abilities. A study on the antioxidant activities of *C*. *racemosum* revealed comparable findings, with higher DPPH scavenging activity than ferric-reducing ability in methanol, ethyl-acetate, and *n*-butanol extracts of the plant’s leaves [54]. Reduction of the ferric ion is dependent on the presence of specific reducing agents within the plant extracts [55,56]. Alternatively, the difference in scavenging activity with ferric-reducing abilities could be due to varied experimental conditions.

### 3.2. Antibacterial Activity

The antibacterial effectiveness of the methanol extracts of *C*. *molle* were determined using the agar well-diffusion technique. This was based on the ability of the extracts to inhibit bacterial growth upon diffusion into the agarose medium [57]. The inhibition of growth was characterized by a clear zone surrounding the well containing the extract [58,59]. Results from the antibacterial assay of this study revealed that the methanol leaf and stem extracts of *C*. *molle* exhibited activity against both Gram-negative *E*. *coli* and Gram-positive *S*. *aureus* bacteria, with significant activity at higher concentrations (5 and 10 mg/mL) where the activity is similar to the antibiotics; 10 µg/mL of gentamicin and streptomycin were demonstrated (Table 3). The extracts’ bacterial inhibition was dose-dependent, possibly attributed to higher quantities of antibacterial-associated phytochemical constituents (e.g., phenolic compounds, flavonoids, and alkaloids) being present at higher extract concentrations [60,61,62]. However, the dose-dependent antibacterial activity was notably unexceptional, insinuating that the responsible phytochemicals did not increase excessively with increasing extract concentration. Plants with antioxidant activities also possess antibacterial properties [19,63,64] due to specific phytocompounds that augment the plant’s antioxidant capabilities; additionally, they are responsible for activity against bacterial pathogens [10,60,65]. Due to their high polarity, there is evidence that methanolic extracts increase the presence of antioxidant- and antibacterial-associated phytochemicals due to possessing a high extraction capacity for these compounds [49,50]. In conjunction with phenolic compounds, flavonoids and alkaloids, tannins, terpenoids, and essential oils are likewise responsible for a plant’s antibacterial effects [7,8,66]. Several studies have reported the presence of these compounds in the leaves and stems of *C*. *molle* [15,19,28,67], substantiating the plant’s antibacterial efficacy. There are several probable modes of action in which these phytochemicals produce their effects. However, a likely mechanism may be the alteration of the bacterial membrane permeability, causing cell destruction and a subsequent decrease in pathogenicity [10].

In the present study, at 10 mg/mL, *S*. *aureus* was more susceptible to the leaf extract than *E*. *coli*. The extract was additionally more effective than the control antibiotic (streptomycin). Gram-positive bacteria generally have a higher susceptibility to antibiotics than Gram-negative bacteria due to the outer peptidoglycan layer of the cell wall being an ineffective antibiotic barrier [10,68]. In contrast, the outer membrane of Gram-negative bacteria contains a periplasmic space harbouring lipopolysaccharides that more effectively prevent the passage of antibiotics into the cell [10,68]. Nevertheless, the leaf extract’s activity against *E*. *coli* was significant, with similar effectiveness as the antibiotic control (gentamicin). The exhibition of significant antibacterial activity against both Gram-negative and Gram-positive strains suggests the latent use of *C*. *molle* extracts as a broad-spectrum antibiotic. An antibacterial evaluation by Cock and Van Vuuren [69] on aqueous and methanolic leaf extracts of *C*. *molle* further revealed the plant’s broad-spectrum nature by exhibiting comparable activity against Gram-negative and Gram-positive strains. The study further reported broad-spectrum capabilities for the leaves of *Combretum cilium*, *Combretum erythrophloeum*, *Combretum erythrophyllum*, *Combretum hereroense,* and *Combretum microphyllum*. Broad-spectrum antibacterial activities were additionally observed in the leaf extracts of *C*. *album* [7] in flavonoids isolated from *C*. *erythrophyllum* [63] and in gold nanoparticles synthesized from leaf extracts of *C*. *erythrophyllum* [70]. Conclusively, *C*. *molle* extracts in the present study were found to be as effective as the standard, synthetic antibiotics. Regardless of the extracts being at significantly higher concentrations than the control antibiotics, it is important to consider that plant extracts are a safer alternative with potentially no toxicity or adverse side effects [1,71,72]. Furthermore, plants are cheaper and more accessible, resulting in better availability and consumption for all communities [71,73]. Moreover, the production and consumption of synthetic antibiotics have been reported to exert detrimental effects on the environment [74,75].

With regards to distinguishing between the plant’s leaf and stem antibacterial effectiveness, it was found that both organs exhibited a similar degree of inhibition of bacterial growth (Table 3). As reported for the plant’s antioxidant activity, comparable antibacterial activities within the extracts of the leaves and stems suggest the presence of similar phytochemicals in both organs. An exception to this was found at extract concentrations of 2.5 mg/mL, where *E*. *coli* was more susceptible to the leaf extract than the stem. Contrasting results were revealed in a study on the ethanol extracts of *C*. *molle*, where leaf and bark extracts produced considerable antibacterial activity against *S*. *aureus*, while seed and stem extracts produced insignificant activities even at high concentrations (100 mg/mL) [76]. These differences may be attributed to variations in locality, climate, season, and extraction methods [44,45]. Furthermore, plant extracts comprise several bioactive constituents, which may be present in minute quantities or subdue the activities of one another [17]. Referring to the findings of this study, it is suggested that both leaf and stem extracts can be used as antibacterial agents due to similar effectiveness. The synergistic usage of both leaves and stems may prevent the exploitation of a single organ, ultimately reducing the risk of destructive harvesting [3,77]. Data from several studies correspond to the findings of *C*. *molle*’s positive antibacterial activity. Asres et al. [18] investigated the plant’s stembark via the disc diffusion technique and found that effective activity was produced against Gram-negative strains; *E*. *coli* (K99, K88, 306, LT37, 872, ROW 7/12, 3:37C, and CD/99/1) and several *Shigella* species were comparable to the control antibiotic ciprofloxacin. Mogashoa et al. [34] investigated the antimicrobial activity of *C*. *molle* leaves via microdilution. The study revealed effective activity from the acetone, ethyl acetate, and dichloromethane extracts against *E*. *faecalis* (ATCC 29212), *S*. *aureus* (ATCC 29213), *E*. *coli* (ATCC 25922), and *P*. *aeruginosa* (ATCC 27853). However, the acetone extracts produced the highest antibacterial effect on all strains, with an average minimum inhibitory concentration of 0.20 mg/mL. Another study revealed that leaves of *C*. *molle* produced moderate activity in comparison to the standard antibiotic chloramphenicol via a microdilution technique, where minimum inhibitory concentrations ranged from 128 μg/mL against *Enterobacter aerogenes* (EA294) to 512 μg/mL against various *E*. *coli* strains (ATCC10536, AG100, AG100A, and W3110) [66]. Ntshanka et al. [19] used the disc-diffusion technique to examine the plant’s antibacterial activities and found significant activity against *E*. *coli* from the plant’s acetone leaf extracts. In addition, numerous studies on the antibacterial activities of other *Combretum* species revealed results that suggest high antibacterial activities present within the genus. Fyhrquist et al. [17] showed that methanol extracts of the *Combretum fragrans* root, as well as the *Combretum padoides* root and stembark, were highly effective against *S*. *aureus* (FOMK). Moreover, *C*. *microphyllum* extracts produced significant activity against *S*. *aureus* (ATCC 29213) and *E*. *coli* (ATCC 25922). A study by Chukwujekwu and Van Staden [33] revealed that a dichloromethane fraction of the leaves of *C*. *edwardsii* was highly effective against *S*. *aureus* (ATCC 11632) when compared to *E*. *coli* (ATCC 25218), with a minimum inhibitory concentration of 0.195 μg/mL. Against *E*. *coli*, the most effective activity was obtained by the ethyl acetate leaf fraction, with a minimum inhibitory concentration of 390 μg/mL. The study further revealed that *C*. *krausii* displayed moderate and weak antibacterial activity against *S*. *aureus* and *E*. *coli*, respectively. Furthermore, cold aqueous, hot aqueous, and ethanol extracts of the leaves of *C*. *album* were effective against *E*. *coli* (MTCC 739) [7]. From all the extracts, leaves extracted in ethanol produced the lowest minimum inhibitory concentration (15 μg/mL).

## 4. Materials and Methods

### 4.1. Plant Material Collection and Extract Preparation

Leaves and stems of *C*. *molle* were collected from a single tree located in a roadside garden on Pitlochry Road (29°49.0985′ S, 30°56.1057′ E), Westville North, Durban, KwaZulu–Natal, South Africa. A voucher specimen was deposited at the Bews Herbarium in the School of Life Sciences, University of KwaZulu–Natal, Pietermaritzburg Campus, with accession No. NU0092543, collected by M. Parusnath (collector no. 1). The leaves and stems were dried in an oven (EcoTherm, Labotec, South Africa) for three weeks at 30 °C. The dried material was ground separately into fine powders using a blender (Russell Hobbs, RHB048, Failsworth, Manchester, UK). Subsequently, 10 g each of the leaf and stem-powdered material was placed into separate 250-mL round-bottom flasks, to which 100 mL of solvent was added. Using a reflux apparatus, the solutions were boiled thrice for three hours, each with an intervening filtration step using a Whatman^®^ No. 1 filter paper. Three different solvents were used; therefore, the reflux process was repeated for each solvent in order of increasing polarity, i.e., hexane, chloroform, and then methanol (Merck, Darmstadt, Germany). The extracted filtrates were stored in hermetic glass bottles at room temperature in the dark.

### 4.2. Antioxidant Assays

For the antioxidant assays, each extract was made up of concentrations of 15, 30, 60, 120, and 240 μg/mL by diluting the sample with its respective solvent (hexane, chloroform, or methanol).

#### 4.2.1. Assay of 2,2′-Diphenyl-1-picrylhydrazyl (DPPH) Radical Scavenging

The antioxidant activity of hexane, chloroform, and methanol leaf and stem extracts of *C*. *molle* was determined using DPPH radical scavenging assay [37,38,39], with modifications as implemented by Akwu et al. [78]. For each extract, 1 mL of its respective concentration and 500 μL of 0.135 mM DPPH (Sigma–Aldrich, Burlington, MA, USA) were added. The solutions were mixed and incubated in the dark at room temperature for 30 min. Following incubation, absorbance was measured at 517 nm using a UV–2600 UV–VIS Spectrophotometer (Shimadzu, Kyoto, Japan), with methanol as a blank. Ascorbic acid (Sigma–Aldrich, Burlington, MA, USA) was used as the standard. All analyses were conducted in triplicate. To determine the DPPH scavenging activity of the extracts, Formula (1) was utilized:

(1) $$\mathrm{DPPH}\;\mathrm{scavenging}\;\mathrm{activity}\;\left(\%\right)=\left(\frac{{\mathrm{Abs}}_{\mathrm{control}}-{\mathrm{Abs}}_{\mathrm{sample}}}{{\mathrm{Abs}}_{\mathrm{control}}}\right)\times \;100;$$

where Abs_control_ was the absorbance of the control (a solution of DPPH with methanol), and Abs_sample_ was the absorbance of the solution of DPPH with the sample (or standard). To evaluate the antioxidant activities of the extracts, the half-maximal inhibitory concentrations or IC_50_ values (the concentration of antioxidants needed to scavenge 50% of the initial DPPH radicals) of each extract were derived from inhibition curves by plotting percentage activity against concentration.

#### 4.2.2. Ferric (Fe^3+^)-Reducing Antioxidant Power (FRAP) Assay

The antioxidant power of hexane, chloroform and methanol leaf, and stem extracts of *C*. *molle* was determined using the FRAP assay [51], with modifications as per an analysis by Akwu et al. [78]. Briefly, 50 µL of 0.2 M sodium phosphate buffer (pH 6.6) (Sigma–Aldrich, USA) and 100 μL of 1% potassium ferricyanide were added to 50 µL of each sample of each concentration. The solutions were mixed and incubated for 30 min at 50 °C in a water bath (Labcon Laboratory Equipment, Krugersdorp, South Africa). Thereafter, 50 μL of 10% trichloroacetic acid was added to each sample, followed by the addition of 50 μL of distilled water and 10 µL of 0.1% iron (III) chloride (FeCl_3_) (Merck, Darmstadt, Germany). The solutions were transferred to a 96-well microtiter plate and absorbance was measured at 700 nm using a Synergy HTX Multi-mode Reader (BioTek Instruments Inc., Winooski, VT, USA). Gallic acid was used as the positive control. All analyses were conducted in triplicate. The results were expressed as a percentage of the absorbance of the extracts relative to gallic acid using Formula (2).

(2) $$\%\;\mathrm{Inhibition}=\left(\frac{{\mathrm{Abs}}_{\mathrm{sample}}}{{\mathrm{Abs}}_{\mathrm{gallic}\;\mathrm{acid}}}\right)\times \;100$$

where Abs_sample_ was the absorbance of the sample and Abs_gallic acid_ was the absorbance of gallic acid (positive control). To evaluate the antioxidant activities of the extracts, IC_50_ values (the concentration of antioxidants required to inhibit oxidation by 50%) of each extract were derived from inhibition curves by plotting percentage inhibition against concentration.

### 4.3. Antibacterial Assay

The antibacterial activity of the methanol leaf and stem extracts of *C*. *molle* was determined using the agar well-diffusion technique as described by [58], with modifications. The assay was performed against clinical strains of Gram-negative *E*. *coli* (ATCC 25922) and Gram-positive *S*. *aureus* (ATCC 25923) bacteria (procured from Prof. J. Lin, Discipline of Microbiology, School of Life Sciences, UKZN, Westville). The previously prepared methanol extracts were dried and maintained at room temperature. Each extract was dissolved in 10% dimethyl sulfoxide (DMSO) to produce concentrations of 0.625, 1.25, 2.5, 5, and 10 mg/mL. The bacterial strains were cultured in Nutrient Broth (Biolab, Pretoria, South Africa) for 18 h at 37 °C. Thereafter, the strains were diluted with Nutrient Broth to an optical density of 0.08–0.1, determined using a UV–2600 UV–VIS Spectrophotometer (Shimadzu, Kyoto, Japan), yielding a final concentration of approximately 1 × 10^8^–1 × 10^9^ bacterial cells/mL. The medium was prepared by mixing 38 g of Mueller–Hinton Agar (MHA) (Biolab, Pretoria, South Africa) in 1 L of distilled water, followed by boiling for 1 min and autoclaving at 121 °C for 20 min. After cooling to room temperature, the medium was poured into sterile 90 mm Petri dishes where it was allowed to set under laminar flow conditions. Each bacterial strain was uniformly swabbed onto the MHA plates using sterile cotton swabs. Sterile micropipette tips were used to punch wells with a diameter of 6 mm into the plates. Subsequently, 100 μL of the methanolic extracts at each concentration was added to the wells. The plates were incubated in an oven (EcoTherm, Labotec, uMhlanga, Durban, South Africa) at 37 °C, and the antibacterial activity was assessed after 18 h by measuring the zones of inhibition (mm). The positive controls used in the assay were antibiotics, viz. 10 μg/mL of gentamicin and 10 μg/mL of streptomycin for the Gram-negative and Gram-positive strains, respectively, whereas 10% DMSO was used as the negative control. The assay was conducted in triplicate.

### 4.4. Statistical Analyses

The data were analyzed using RStudio 2022.02.1+461 for Windows (Boston, MA, USA). A one-way analysis of variance (ANOVA) was used to test for inter-treatment differences in antioxidant and antibacterial activity. Means for all analyses of variance were separated using a Tukey post-hoc test. All differences were considered significant at the 0.05 level.

## 5. Conclusions

Assays of DPPH radical scavenging and agar well-diffusion revealed that the methanol leaf and stem extracts of *C*. *molle* were highly effective as antioxidant and antibacterial agents, respectively. The observed activity was significantly higher than the controls, suggesting a strong hydrogen donating capacity and a strong ability to inhibit bacterial growth. Both leaf and stem extracts exhibited similar antioxidant and antibacterial activities, proposing their synergistic use. However, the extracts’ poor ability to reduce ferric ions, as shown by the data obtained from the FRAP assay, limits knowledge on the plant’s oxidant reducing capabilities. Therefore, it is recommended that the assay be performed on the plant’s isolated phytocompounds. We propose that these extracts and their bioactive compounds may produce optimum alleviation of symptoms and disease related to oxidative stress and bacterial infection, and may, hence, be used in drug development. These findings additionally validate the use of this species in traditional medicine and potentially provide disadvantaged communities with an alternative source of medicinal treatment. However, assays about the toxicity of *C. molle* extracts should be performed to assure its validation and safe use. Additionally, it is recommended that further research be conducted on the identification and isolation of the specific phytochemical constituents responsible for the plant’s antioxidant and antibacterial capabilities.

## Figures and Tables

**Table 1:** IC_50_ values (µg/mL) of the DPPH radical scavenging activities of leaf and stem hexane, chloroform and methanol extracts of *C*. *molle*.

Extract	Solvent	IC_50_ (µg/mL)
Leaf	Hexane	42.57 ± 9.88 ^b^
	Chloroform	118.12 ± 28.07 ^b^
	Methanol	1.52 × 10^−7^ ± 2.93 × 10^−8 b^
Stem	Hexane	1373.30 ± 479.44 ^a^
	Chloroform	0.22 ± 0.09 ^b^
	Methanol	2.46 × 10^−14^ ± 8.53 × 10^−15 b^
Ascorbic acid (standard)		0.01 ± 0.00 ^b^

Values presented are means ± standard deviation of three replicates. Values followed by different letters are significantly different at *p* < 0.05.

**Table 2:** IC_50_ values (µg/mL) of the ferric-reducing antioxidant power of the leaf and stem hexane, chloroform, and methanol extracts of *C*. *molle*.

Extract	Solvent	IC_50_ (µg/mL)
Leaf	Hexane	6292.55 ± 795.31 ^cd^
	Chloroform	2948.11 ± 868.55 ^cd^
	Methanol	663.77 ± 74.71 ^bd^
Stem	Hexane	8358.53 ± 5514.41 ^ac^
	Chloroform	2602.06 ± 1856.93 ^cd^
	Methanol	1368.11 ± 424.54 ^cd^
Gallic acid (control)		0.53 ± 0.16 ^bd^

Values presented are means ± standard deviation of three replicates. Values followed by different letters are significantly different at *p* < 0.05.

**Table 3:** Zones of inhibition (mm) of the methanolic leaf and stem extracts of *C*. *molle* against Gram-negative *E*. *coli* and Gram-positive *S*. *aureus* bacteria.

Bacterial Strain	Extract Concentration (mg/mL)					Positive Control (10 µg/mL)
	0.625	1.25	2.5	5	10	
Leaf						
*E*. *coli*	6.67 ± 0.24 *	7.33 ± 1.18 *	7.50 ± 1.08 *^†^	10.00 ± 1.22	12.00 ± 0.00 ^§^	12.33 ± 0.47
*S*. *aureus*	7.00 ± 0.00 *	7.00 ± 0.00 *	8.67 ± 0.24 *	11.00 ± 0.00	14.50 ± 1.08 ^§^	12.33 ± 1.25
Stem						
*E*. *coli*	6.33 ± 0.24 *	7.00 ± 0.00 *	7.00 ± 0.41 *^†^	9.67 ± 0.47*	11.83 ± 0.62	12.67 ± 0.47
*S*. *aureus*	7.17 ± 0.62 *	7.17 ± 0.62 *	7.50 ± 1.08 *	10.33 ± 0.47*	12.33 ± 0.47	13.33 ± 0.94

Values presented are means ± standard deviation of three replicates. *E. coli* = *Escherichia coli* (ATCC 25922); *S*. *aureus = Staphylococcus aureus* (ATCC 25923); positive controls: 10 µg/mL of gentamicin (Gram-negative antibiotic) and 10 µg/mL of streptomycin (Gram-positive antibiotic); * indicates significant differences in inhibition relative to the respective control; † indicates significant differences in inhibition between different extracts against the same strain; § indicates significant differences in inhibition between the same extract against different strains; (*p* < 0.05).

## Data Availability

All data are presented in the article.

## References

[1] Wink M. (2015). Modes of action of herbal medicines and plant secondary metabolites. Medicines.

[2] Jalali A., Dabaghian F., Akbrialiabad H., Foroughinia F., Zarshenas M.M. (2021). A pharmacology-based comprehensive review on medicinal plants and phytoactive constituents possibly effective in the management of COVID-19. Phytother. Res..

[3] Van Wyk A., Prinsloo G. (2018). Medicinal plant harvesting, sustainability and cultivation in South Africa. Biol. Conserv..

[4] Ozioma E.-O.J., Chinwe O.A.N. (2019). Herbal medicines in African traditional medicine. Herb. Med..

[5] Karimi A., Majlesi M., Rafieian-Kopaei M. (2015). Herbal versus synthetic drugs; beliefs and facts. J. Nephropharmacol..

[6] Farooq S., Ngaini Z. (2021). Natural and synthetic drugs as potential treatment for coronavirus disease 2019 (COVID-2019). Chem. Afr..

[7] Burman S., Bhattacharya K., Mukherjee D., Chandra G. (2018). Antibacterial efficacy of leaf extracts of *Combretum album* Pers. against some pathogenic bacteria. BMC Complement Altern. Med..

[8] Kengne I.C., Feugap L.D.T., Njouendou A.J., Ngnokam C.D.J., Djamalladine M.D., Ngnokam D., Voutquenne-Nazabadioko L., Tamokou J.-D.-D. (2021). Antibacterial, antifungal and antioxidant activities of whole plant chemical constituents of *Rumex abyssinicus*. BMC Complement Altern. Med..

[9] Daria S., Islam M.R. (2022). Indiscriminate Use of Antibiotics for COVID-19 Treatment in South Asian Countries is a Threat for Future Pandemics Due to Antibiotic Resistance. Clin. Pathol..

[10] Safari M., Ahmady-Asbchin S. (2019). Evaluation of antioxidant and antibacterial activities of methanolic extract of medlar (*Mespilus germanica* L.) leaves. Biotechnol. Biotechnol. Equip..

[11] Les F., Cásedas G., López V. (2021). Bioactivity of medicinal plants and extracts. Biology.

[12] Smith H.H. (1928). Ethnobotany of the Meskwaki Indians.

[13] Ehlert E. (1968). A History of Medicine in the Early Years.

[14] Rodgers C., Verotta L. (1996). Chemistry and biological properties of the African Combretaceae. Chemistry, Biological and Pharmacological Properties of African Medicinal Plants.

[15] Hamza R.Z., Al-Baqami N.M., Khojah E., Mansour A.M., Al-Motaani S.E., Al-Salmi F.A., El-Megharbel S.M. (2021). Possible antioxidant and antidiabetic effects of *Combretum molle* extract in a diabetes mellitus experimental model in male rats. Nat. Prod. Commun..

[16] Estella O.U., William A.C., Patrick O., Ikenna C., Mba T., Obinna O., Ginikachukwu U. (2022). Evaluation of the analgesic and antipyretic activity of methanol extract of *Combretum bauchiense* Hutch & Dalziel (Combretaceae) leaves. Phytomed. Plus.

[17] Fyhrquist P., Mwasumbi L., Hæggström C.-A., Vuorela H., Hiltunen R., Vuorela P. (2002). Ethnobotanical and antimicrobial investigation on some species of *Terminalia* and *Combretum* (Combretaceae) growing in Tanzania. J. Ethnopharmacol..

[18] Asres K., Mazumder A., Bucar F. (2006). Antibacterial and antifungal activities of extracts of *Combretum molle*. Ethiop. Med. J..

[19] Ntshanka N.M., Ejidike I.P., Mthunzi F.M., Moloto M.J., Mubiayi K.P. (2020). Investigation into the phytochemical profile, antioxidant and antibacterial potentials of *Combretum molle* and *Acacia mearnsii* leaf parts. Biomed. Pharmacol. J..

[20] Asres K., Bucar F., Knauder E., Yardley V., Kendrick H., Croft S. (2001). In vitro antiprotozoal activity of extract and compounds from the stem bark of *Combretum molle*. Phytother. Res..

[21] Ahmed B., Al-Howiriny T., Passreiter C., Mossa J. (2004). Combretene-A and B: Two new triterpenes from *Combretum molle*. Pharm. Biol..

[22] Pegel K.H., Rogers C.B. (1985). The characterisation of mollic acid 3β-D-xyloside and its genuine aglycone mollic acid, two novel 1α-hydroxycycloartenoids from *Combretum molle*. J. Chem. Soc. Perkin Trans..

[23] Ponou B.K., Barboni L., Teponno R.B., Mbiantcha M., Nguelefack T.B., Park H.-J., Lee K.-T., Tapondjou L.A. (2008). Polyhydroxyoleanane-type triterpenoids from *Combretum molle* and their anti-inflammatory activity. Phytochem. Lett..

[24] Masoko P., Picard J., Eloff J. (2005). Antifungal activities of six south African *Terminalia* species (Combretaceae). J. Ethnopharmacol..

[25] Gruz J., Ayaz F.A., Torun H., Strnad M. (2011). Phenolic acid content and radical scavenging activity of extracts from medlar (*Mespilus germanica* L.) fruit at different stages of ripening. Food Chem..

[26] Muhtadi M., Wiyono A.A.F. (2020). Testing antioxidant activity of *Plumeria Alba* and *Plumeria Rubra* ethanolic extracts using DPPH and Frap methods and determining their total flavonoid and phenolic levels. J. Nutrac. Herb. Med..

[27] Palipoch S. (2013). A review of oxidative stress in acute kidney injury: Protective role of medicinal plants-derived antioxidants. Afr. J. Tradit. Complement. Altern. Med..

[28] Koevi K.-K.A., Millogo V., Fokou J.B.H., Sarr A., Ouedraogo G.A., Bassene E. (2015). Phytochemical analysis and antioxidant activities of *Combretum molle* and *Pericopsis laxiflora*. Int. J. Biol. Chem. Sci..

[29] Babatunde S., Moyinoluwa O., Oluwatosin A., Eigege W., Shreyans J. (2014). Bioguided isolation of an antioxidant compound from *Combretum racemosum* P. Beav leaf. J. Biol. Chem. Sci..

[30] Bhuiya N.M.A. (2020). Investigation on antioxidant and antimicrobial properties of methanolic extract of *Combretum indicum* leaf. Int. J. Green Pharm..

[31] Rajalingam D., Varadharajan R., Palani S. (2016). Evaluation of hepatoprotective and antioxidant effect of *Combretum albidum* G. don against CCl4 induced hepatotoxicity in rats. Int. J. Pharm. Pharm. Sci..

[32] Stanković N., Mihajilov-Krstev T., Zlatković B., Stankov-Jovanović V., Mitić V., Jović J., Čomić L., Kocić B., Bernstein N. (2016). Antibacterial and antioxidant activity of traditional medicinal plants from the Balkan Peninsula. NJAS -Wagening. J. Life Sci..

[33] Chukwujekwu J.C., Van Staden J. (2016). *In vitro* antibacterial activity of *Combretum edwardsii*, *Combretum krausii*, and *Maytenus nemorosa* and their synergistic effects in combination with antibiotics. Fron. Pharmacol..

[34] Mogashoa M., Masoko P., Eloff J. (2019). Different *Combretum molle* (Combretaceae) leaf extracts contain several different antifungal and antibacterial compounds. S. Afr. J. Bot..

[35] Mu J., Uehara T., Li J., Furuno T. (2004). Identification and evaluation of antioxidant activities of bamboo extracts. For. Stud. China..

[36] Agbor A.M., Naidoo S. (2011). Knowledge and practice of traditional healers in oral health in the Bui Division, Cameroon. J. Ethnobiol. Ethnomedicine..

[37] Blois M.S. (1958). Antioxidant determinations by the use of a stable free radical. Nature.

[38] Braca A., Sortino C., Politi M., Morelli I., Mendez J. (2002). Antioxidant activity of flavonoids from *Licania licaniaeflora*. J. Ethnopharmacol..

[39] Mishra K., Ojha H., Chaudhury N.K. (2012). Estimation of antiradical properties of antioxidants using DPPH assay: A critical review and results. Food. Chem..

[40] Dumanović J., Nepovimova E., Natić M., Kuča K., Jaćević V. (2021). The significance of reactive oxygen species and antioxidant defense system in plants: A concise overview. Front. Plant Sci..

[41] Rademan S., Anantharaju P.G., Madhunapantula S.V., Lall N. (2019). The anti-proliferative and antioxidant activity of four indigenous South African plants. Afr. J. Tradit. Complement. Altern. Med..

[42] Njoya E.M. (2021). Medicinal plants, antioxidant potential, and cancer. Cancer.

[43] Mulaw T., Wubetu M., Dessie B., Demeke G., Molla Y. (2019). Evaluation of antimalarial activity of the 80% methanolic stem bark extract of *Combretum molle* against *Plasmodium berghei* in mice. J. Evid.-Based Integr. Med..

[44] Dutta S., Ray S. (2020). Comparative assessment of total phenolic content and *in vitro* antioxidant activities of bark and leaf methanolic extracts of *Manilkara hexandra* (Roxb.) Dubard. J. King Saud Univ. Sci..

[45] Naidoo C.M., Naidoo Y., Dewir Y.H., Singh M., Daniels A.N., El-Ramady H. (2022). *In Vitro* investigation of the antioxidant and cytotoxic potential of *Tabernaemontana ventricosa* hochst. Ex A. DC. leaf, stem, and latex extracts. Horticulturae.

[46] Aderogba M., Kgatle D.T., McGaw L.J., Eloff J.N. (2012). Isolation of antioxidant constituents from *Combretum apiculatum* subsp.. apiculatum. S. Afr. J. Bot..

[47] Manga F.N., El Khattabi C., Fontaine J., Berkenboom G., Duez P., Nzunzu J.L., Pochet S. (2012). Vascular effects and antioxidant activity of two *Combretum* species from Democratic Republic of Congo. J. Ethnopharmacol..

[48] Kpemissi M., Eklu-Gadegbeku K., Veerapur V.P., Potârniche A.-V., Adi K., Vijayakumar S., Banakar S.M., Thimmaiah N., Metowogo K., Aklikokou K. (2019). Antioxidant and nephroprotection activities of *Combretum micranthum*: A phytochemical, in-vitro and ex-vivo studies. Heliyon.

[49] Henkel S., Misuraca M.C., Troselj P., Davidson J., Hunter C.A. (2018). Polarisation effects on the solvation properties of alcohols. Chem. Sci.

[50] Roopashree K., Naik D. (2019). Advanced method of secondary metabolite extraction and quality analysis. J. Pharmacogn. Phytochem..

[51] Benzie I.F., Strain J.J. (1996). The ferric reducing ability of plasma (FRAP) as a measure of “antioxidant power”: The FRAP assay. Anal. Biochem..

[52] Fernandes R.d.P., Trindade M., Tonin F., Lima C., Pugine S., Munekata P., Lorenzo J., De Melo M. (2016). Evaluation of antioxidant capacity of 13 plant extracts by three different methods: Cluster analyses applied for selection of the natural extracts with higher antioxidant capacity to replace synthetic antioxidant in lamb burgers. J. Food Sci. Technol..

[53] Bashir M. (2022). Ameliorative Potential of Ethyl Acetate and Aqueous Fractions of Methanol Leaf Extract of *Combretum micranthum* against Free Radicals. Saudi J. Med. Pharm. Sci..

[54] Amos-Tautua B., Oluwafemi O., Ajileye O., Alayande K., Olawuni I., Bamidele F., Onigbinde A., Songca S. (2017). Antimicrobial, antioxidant activities *in vitro* and polyphenol contents of the leaf extract of a versatile medicinal plant. Asian J. Appl. Sci..

[55] Müller F., Rapp J., Hacker A.-L., Feith A., Takors R., Blombach B. (2020). CO_2_/HCO_3_^−^ accelerates iron reduction through phenolic compounds. MBio.

[56] Chirumamilla P., Taduri S. (2022). Assessment of in vitro anti-inflammatory, antioxidant and antidiabetic activities of *Solanum khasianum* Clarke. Vegetos.

[57] Bonev B., Hooper J., Parisot J. (2008). Principles of assessing bacterial susceptibility to antibiotics using the agar diffusion method. J. Antimicrob. Chemother..

[58] Perez C. (1990). Antibiotic assay by agar-well diffusion method. Acta Biol. Med. Exp..

[59] Manandhar S., Luitel S., Dahal R.K. (2019). *In vitro* antimicrobial activity of some medicinal plants against human pathogenic bacteria. J. Trop. Med..

[60] Cushnie T.T., Lamb A.J. (2005). Antimicrobial activity of flavonoids. Int. J. Antimicrob. Agents.

[61] Rempe C.S., Burris K.P., Lenaghan S.C., Stewart Jr C.N. (2017). The potential of systems biology to discover antibacterial mechanisms of plant phenolics. Front. Microbiol..

[62] Adamski Z., Blythe L.L., Milella L., Bufo S.A. (2020). Biological activities of alkaloids: From toxicology to pharmacology. Toxins.

[63] Martini N., Katerere D., Eloff J. (2004). Biological activity of five antibacterial flavonoids from *Combretum erythrophyllum* (Combretaceae). J. Ethnopharmacol..

[64] Marquardt P., Seide R., Vissiennon C., Schubert A., Birkemeyer C., Ahyi V., Fester K. (2020). Phytochemical characterization and *in vitro* anti-inflammatory, antioxidant and antimicrobial activity of *Combretum collinum* Fresen leaves extracts from Benin. Molecules.

[65] Mitani T., Ota K., Inaba N., Kishida K., Koyama H.A. (2018). Antimicrobial activity of the phenolic compounds of *Prunus mume* against Enterobacteria. Biol. Pharm. Bull..

[66] Fankam A.G., Kuiate J.R., Kuete V. (2015). Antibacterial and antibiotic resistance modifying activity of the extracts from *Allanblackia gabonensis*, *Combretum molle* and *Gladiolus quartinianus* against Gram-negative bacteria including multi-drug resistant phenotypes. BMC Complement Altern. Med..

[67] Saidu T., Abdullahi M. (2011). Phytochemical determinations and antibacterial activities of the leaf extracts of *Combretum molle* and *Gossypium arboretum*. Bayero J. Pure Appl. Sci..

[68] Nikaido H. (2003). Molecular basis of bacterial outer membrane permeability revisited. Microbiol. Mol. Biol. Rev..

[69] Cock I., Van Vuuren S. (2015). A comparison of the antimicrobial activity and toxicity of six *Combretum* and two *Terminalia* species from Southern Africa. Pharmacogn. Mag..

[70] Fanoro O.T., Parani S., Maluleke R., Lebepe T.C., Varghese J.R., Mavumengwana V., Oluwafemi O.S. (2021). Facile Green, Room-Temperature Synthesis of Gold Nanoparticles Using *Combretum erythrophyllum* Leaf Extract: Antibacterial and Cell Viability Studies against Normal and Cancerous Cells. Antibiotics.

[71] Oguntibeju O.O. (2018). Medicinal plants with anti-inflammatory activities from selected countries and regions of Africa. J. Inflamm. Res..

[72] Bungau S., Tit D.M., Behl T., Aleya L., Zaha D.C. (2021). Aspects of excessive antibiotic consumption and environmental influences correlated with the occurrence of resistance to antimicrobial agents. Curr. Opin. Environ. Sci. Health..

[73] Abdullahi A.A. (2011). Trends and challenges of traditional medicine in Africa. Afr. J. Tradit Complement. Altern. Med..

[74] Polianciuc S.I., Gurzău A.E., Kiss B., Ştefan M.G., Loghin F. (2020). Antibiotics in the environment: Causes and consequences. Med. Pharm. Rep..

[75] Mathur P., Sanyal D., Callahan D.L., Conlan X.A., Pfeffer F.M. (2021). Treatment technologies to mitigate the harmful effects of recalcitrant fluoroquinolone antibiotics on the environment and human health. Environ. Pollut..

[76] Regassa F., Araya M. (2012). *In vitro* antimicrobial activity of *Combretum molle* (Combretaceae) against *Staphylococcus aureus* and *Streptococcus agalactiae* isolated from crossbred dairy cows with clinical mastitis. Trop. Anim. Health Prod..

[77] Reid A.-M., Oosthuizen C.B., Fibrich B.D., Twilley D., Lambrechts I.A., de Canha M.N., Rademan S., Lall N. (2018). Traditional medicine: The ancient roots of modern practice. Medicinal Plants for Holistic Health and Well-Being.

[78] Akwu N., Naidoo Y., Singh M., Nundkumar N., Lin J. (2019). Phytochemical screening, in vitro evaluation of the antimicrobial, antioxidant and cytotoxicity potentials of *Grewia lasiocarpa* E. Mey. ex Harv. S. Afr. J. Bot..

